# Midwifery practice in Mongolia: Policy implications for accelerating care delivery transformation

**DOI:** 10.18332/ejm/184209

**Published:** 2024-06-03

**Authors:** Baigalmaa Dovdon, Nigel McCarley, Jinhyun Kim

**Affiliations:** 1 School of Nursing, Mongolian National University of Medical Sciences, Ulaanbaatar, Mongolia; 2 Ulster University, Belfast, United Kingdom; 3 College of Nursing, Seoul National University, Seoul, Korea

**Keywords:** shortage of midwives, midwifery leadership, quality of midwifery care, post-graduate training, midwifery education

## Abstract

Midwives can play a significant role in reducing maternal and neonatal mortality rates as well as in improving maternal and perinatal health outcomes in low- and middle-income countries such as Mongolia. However, the shortage of midwives in Mongolia is severe. Despite the evidence indicating numerous challenges associated with substandard midwifery education and practice in Mongolia, there is a need for policy recommendations to accelerate the improvement of midwifery care delivery in the country. Therefore, we identified three main topics as key issues in Mongolian midwifery care: 1) the current training and service delivery in midwifery; 2) the potential for the development of the midwifery role; and 3) content requirements for the postgraduate certificate in midwifery. The World Health Organization report made forty recommendations to enhance the midwife's role to professional status. However, the absence of senior nursing/midwifery leadership in the Ministry of Health in Mongolia has hindered the implementation of the recommendations. To strengthen midwifery at a national level, it is imperative to make multilevel investments in supporting educators and clinical practitioners to ensure the delivery of high-quality midwifery care. This article represents an initial in-depth exploration of issues in Mongolian midwifery practice, with the aim of providing suggestions for practical avenues for enhancing midwifery care in Mongolia.

## INTRODUCTION

Midwifery has been practiced for centuries and provides care to women throughout pregnancy and childbirth. Midwifery care has been a focus of the Mongolian government to improve maternal–child outcomes. Mongolia is the least densely populated country in the world with a population density of 2.1 people/km^2^. In addition, approximately 30% of the people are nomadic, presenting a unique challenge for the delivery of maternal care^1^. In the last decade, Mongolia has implemented significant positive steps regarding maternal care, such as the National Program ‘Maternal, Child, and Reproductive Health 2017-2021’. As a result, at the end of August 2019, the nationwide Maternal Mortality Ratio (MMR) had decreased substantially from the 2016 baseline level of 48.6 per 100000 live births to 21.2, lower than the target of 25. The rate of neonatal mortality had also decreased from the 2016 baseline of 9.2 per 1000 live births to 8.1 (the target is 8.5). By the end of 2019, the maternal mortality rate reached 23.0%, well within the criteria set by the National Program^2^.

Although maternal mortality has been decreasing in Mongolia, it has increased in the last two years. By the end of the third quarter of 2021, 68 mothers had died, and the maternal mortality rate had risen to 94.9 per 100000 live births, triple that of the same period of the previous year^3^. The COVID-19 epidemic in the country contributed in part to the increase in maternal mortality. However, other factors such as the availability of human resources, workloads, insufficient policy implementation, poor quality of medical care, and lack of beds, also had a corresponding effect^4,5^.

To improve maternal health, it is critical to identify and address potential and substantial barriers both at the health system and societal level that limit access to adequate-quality maternal health services^6^. Regrettably, locating this information within the existing literature is an arduous task, posing a significant barrier to enhancing the quality of midwifery care in Mongolia through the identification and enhancement of areas in need of improvement in midwifery care delivery in the country.

According to the World Health Organization (WHO), the Ending Preventable Target aims to reduce the global maternal mortality ratio by 2030, by adopting the Sustainable Development Goals (SDG) 3.1 target to reduce global MMR to <70 per 100000 live births by 2030^7^. However, the critical shortage of healthcare workers, coupled with the growing disease burden, poses a significant threat to the sustainability of health systems in low- and middle-income countries. This situation impedes achieving universal health coverage, as outlined in SDGs, in these nations^8^.

Also, the vast scale of the Mongolian landscape (about 1.5 million km^2^) dictates that many women must travel long distances to receive maternal care in provincial health facilities, a problem mitigated to some extent by ‘maternity waiting (rest) homes’ established under Ministerial Order A/318^9^. The aforementioned law also allows women from ‘the countryside’ to stay in a maternal waiting (rest) home before delivery, thus, maternal waiting homes have become a critical component of the healthcare referral system.

## COMMENTARY

Midwives can play a significant role in reducing maternal and neonatal mortality rates as well as in improving maternal and perinatal health outcomes in low- and middle-income countries. Thus, around the world, about 1 million midwives will be needed by 2035 to save an estimated 4.3 million lives per year^10^.

However, the shortage of midwives in Mongolia is serious. Specifically, as of 2019, there were 37.3 physicians, 40.4 nurses, and 3.3 midwives per 10000 people in Mongolia. A total of 1051 midwives work in the Mongolian health sector, representing 10% of approximately 12500 nurses^2^. Internationally, a midwife aids 4–6 pregnant mothers on average, but one Mongolian midwife provides maternity care to 15 mothers. That is, Mongolia’s midwifery workforce is nearly three times worse than international standards.

The WHO^11^ report contained 40 recommendations relating to midwifery services, each made to move the profession towards the International Confederation of Midwives (ICM) definition and scope of practice to enhance the role of midwives to a global standard. Yet, the implementation of the 40 recommendations outlined in the 2007 WHO^11^ report remains to be accomplished in Mongolia.

To address the gaps, this article, based on the WHO’s^11^ report on midwifery care in Mongolia, aims to provide a comprehensive and focused review of: 1) The current training and service delivery in midwifery; 2) The potential for the development of the midwifery role; and 3) Content requirements for the postgraduate certificate in midwifery.

### Current training and service delivery in midwifery

*Training*

High-quality midwifery education is crucial to equip midwives with the necessary skills to provide effective Sexual, Reproductive, Maternal, Newborn, and Adolescent Healthcare (SRMNAH) and an acceptable way to reduce maternal and newborn morbidity and mortality rates. Programs that meet global midwifery standards, including duration of the program, quality clinical skills education, clinical experiences, and support to ensure that the full scope of midwifery practice is achieved, are more likely to provide quality education and training^12,13^.

In Mongolia, midwives face several challenges in their quest for education. Historically, general nurses could undertake a 3-month ‘specialist’ training in midwifery after completing the Diploma in Nursing program. However, the program that provided the skills necessary for nurses to work as maternal nurses has been discontinued. Additionally, midwifery education for midwives was suspended from 1993 to 2003, and midwifery as a practice was suspended from 2000 to 2017. Currently, only five service providers deliver training for midwives in Mongolia. They offer two undergraduate training programs for midwives comprising a 3-year program leading to the award of a Diploma in Midwifery and a 4-year program (which was introduced in 2011) leading to the award of Bachelor of Science in Midwifery. Above all, no advanced, postgraduate training programs in midwifery exist in Mongolia. The requirements to be a midwife mandate that a nurse must attend a postgraduate training program to secure the 15 points (3 credit points each year between licensing periods) necessary for re-registration every five years. However, the courses attended are not specific to midwifery. It is questionable whether the postgraduate training program can ensure the midwifery practice’s up-to-date skills and knowledge necessary to satisfy maternal and neonatal care needs in Mongolia.

*Service delivery*

In many parts of the world, midwives are the primary care providers for childbearing women^14^. However, there are considerable variations in the organization of midwifery services and the education and role of midwives.

The ICM^15^ defines the role as follows: ‘A midwife is a person who has completed a midwifery education program recognized in the country offering the course based on the ICM Essential Competencies for Basic Midwifery Practice and the framework of the ICM Global Standards for Midwifery Education; who has acquired the requisite qualifications to be registered and/or legally licensed to practice midwifery and use the title ‘midwife’; and who demonstrates competency in the practice of midwifery’.

ICM^15^ identifies the scope of the practice of a midwife as follows: ‘The midwife is recognized as a responsible and accountable professional, who works in partnership with women to provide the necessary support, care, and advice during pregnancy, labor, and the postpartum period, to perform births on the midwife’s responsibility and provide care for the newborn and the infant. This care includes preventive measures, promotion of normal birth, detection of complications in the mother and child, access to medical care or other appropriate assistance, and the implementation of emergency measures’.

A literature review demonstrates that obstetrician-led care (medical model) and midwifery-led care are the two overarching models of services in maternal care. However, the models are not mutually exclusive, with midwives and medical staff working together (shared care) or in isolation, as dictated by the needs of the pregnant woman or (as in Mongolia) by the organizational structure of services. Nevertheless, there are significant differences between the two models, including variations in philosophy, the provider–patient relationship, the use of obstetric interventions during labor, and the overall goals of care^16,17^.

The difference in philosophy and approach underpins the reported outcomes, with the significant benefits of midwifery-led care well-documented. For example, Sandall et al.^14^ conducted a comparative study involving more than 17000 women and concluded that midwifery-led care offers several benefits without adverse effects, including reduced use of analgesia, fewer episiotomies, and fewer instrumental births. A comparable multinational study conducted by Cragin and Kennedy^18^ found that midwives experienced more favorable care processes (reduced reliance on technology and interventions) without any difference in neonatal outcomes, even after accounting for pre-existing risk factors. The findings are consistent with those of Rooks^16^.

The midwifery model has advantages for many women by minimizing unnecessary interventions during labor, thus supporting a natural process. In addition, it addresses needs that are frequently underserved by the medical management model. The midwifery model also encourages more ‘women-centered’ care than the medical model^19^ and promotes the ‘normalization’ of childbirth^20,21^, a perspective endorsed in a national report on the future of maternity care in the United Kingdom^22,23^. Interviews with both academic and service staff involved in midwifery indicate that both models exist in the Mongolian maternity service but in an apparent dichotomy of services between Ulaanbaatar and the countryside. In Ulaanbaatar, it is evident that the biomedical model is prevalent with the role described as ‘no more than a nursing role’, a role that can assist the obstetrician. The job description for midwives in Ulaanbaatar indicates the full range of activities associated with the role of a midwife, but interviewees reported that midwives’ skills were underutilized. Further, a demotivating work environment exists for the midwives, as they do not perform even the basic midwifery functions.

However, in the countryside, midwifery-led care is more prevalent and their role more closely reflects the international definition. In particular, the role of the Feldsher midwife encapsulates the essence of the autonomous midwifery role most closely. In many cases, the midwife will manage the pregnancy, providing all antenatal care with limited intervention/support from an obstetrician. At times, the midwives are also allowed to work independently from an obstetrician. Reports indicate that midwives in rural areas provide antenatal care per Ministerial Order^9^ as follows:

1. Normal pregnancy: six antenatal visits plus delivery;
2. Moderate risk pregnancy: eight antenatal visits plus delivery; and
3. High-risk pregnancy: transfer to tertiary services and managed by an obstetrician.

Also, the rural midwife is often responsible for identifying clinical events that require referral/transfer to tertiary hospitals.

Here, there seems to be a need for additional discussion on the phenomenon of the divided service delivery model in midwifery care between Ulaanbaatar and the countryside in Mongolia, exploring why it is happening and how it negatively impacts maternal care in Mongolian midwifery.

### Potential for the development of the midwifery role

Advanced practice is based on four fundamental components: clinical practice, leadership and management, education, and research^24^. The WHO^11^ report highlighted the need for continuous education for midwives in Mongolia and the implementation of postgraduate education. However, this recommendation was in the context of the other service development proposals in the report, including, most importantly, the further development of the role of the midwife.

To secure this objective, the WHO^11^ recommended: ‘creating a position of Chief Midwife to sit side-by-side with the Chief Nurse’ (Recommendation 38). In addition, the report states that the role of the Chief Nurse is ‘to assist with policy direction, technical advice, and support in the Ministry of Health’. A Nursing Cohort (including a senior midwife) was also recommended to be established in the Ministry of Health to ensure highly effective leadership of the midwifery profession in Mongolia^25^.

The evidence indicates that, in the absence of meaningful leadership of the midwifery profession at the Ministerial level, midwifery services in Mongolia will continue to be dominated by the medical model and the real potential for developing the midwifery role will continue to be curtailed.

The State of the World’s Midwifery 2021 report provides comprehensive guidance to support the development of midwives and also suggests that investments should be made in midwife-led models of care, midwifery leadership and governance, and high-quality education and training of midwives. By investing in these areas, we can optimize the roles of midwives and ensure high-caliber care for all^10^.

The shortage of leadership directly impacts consideration for developing a postgraduate certificate in midwifery. Postgraduate training is designed to build on undergraduate learning to prepare staff for new roles requiring more advanced knowledge or skills, or to prepare the team to do the same job but with greater knowledge, skill, and expertise.

Internationally, the development of midwifery practice provides for the expansion of roles and skills in managerial, academic, and especially in clinical areas.

*Clinical service provision*

Midwives can choose to develop specialist knowledge on particular aspects of service, e.g. prescribing medications, infant feeding, bereavement counseling, etc. With further training, midwives can move into more advanced practitioner roles, e.g. in the field of neonatal care. The Nursing and Midwifery Board states that ‘Advanced midwifery practice requires autonomous, experienced professionals who are competent, accountable, and responsible for their practice’^24^. Ultimately, positions such as consultant midwife are available for those who attain higher levels of education (for example, MSc and PhD).

Internationally, it is common to find midwives who have assumed a ‘specialist’ clinical role. It is self-evident that such positions could make a significant contribution within the main tertiary hospitals in Mongolia. However, as previously indicated, maternity care within tertiary hospitals is dominated by the medical model in which midwives perform ‘assistant’ roles for obstetricians.

An organizational culture dominated by the medical model presents a significant hurdle to role development in midwifery. Indeed, the expansion of the midwife’s role can be seen as a significant shift in the field of obstetrics. Established obstetricians, who are accustomed to traditional roles and responsibilities, might perceive this as an intrusion into their professional domain. This could lead to resistance towards the necessary changes that come with the evolution of healthcare practices. However, it is important to note that the intention behind the development of the midwife’s role is to enhance patient care and not to diminish the role of obstetricians. The collaboration between midwives and obstetricians can potentially lead to a more holistic and patient-centered approach to care. Further, plans for role development require planning for the midwifery profession. Optimization of the role of midwives through postgraduate education would dictate the need to establish a clinical career path that encompasses ‘advanced’ functions.

Typically, the nursing and midwifery professions mirror the sub-specializations within medicine. Discussions are currently taking place between the United Nations Population Fund and the University of Sydney to revise and introduce a new medical training program. The impact on their complementary roles in the longer term may occur; however, the effect on organizational culture may not occur for many years, if at all.

The present limited function of the midwife and the absence of certified midwife teachers in Mongolia, mean that it would be difficult to ensure essential clinical practice and supervision within the hospital. Without mandates established by the ‘Nursing Cohort’, it is considered that development of the midwifery role in Ulaanbaatar will be unlikely in the foreseeable future.

As previously indicated, the midwife’s role in the countryside more closely reflects the international definition of the midwifery role, with midwifery-led care more prevalent and the ICM ‘scope of practice’ reflected in activities. The role of the Feldsher midwife is indeed an ‘advanced’ role, although there is no ‘postgraduate’ training for this cohort.

The expansive geographical location of midwives in rural areas is another limiting factor. Getting midwives to relocate to Ulaanbaatar for six months of training would present personal and geographical difficulties. There would also be a need to ensure that midwifery cover is provided during their absence when attending a course. The development of a planned rotational system for midwives would give the opportunity; however, establishing such a plan would require the current Ulaanbaatar staff to be prepared to assume positions in the most isolated parts of the country.

The compliance with the National E-Health Strategy states that ‘the E-Health Strategy has defined priority action areas for e-health in the field of developing the health workforce, improving the quality of healthcare services through the use of telemedicine and other applications of e-health’^26^. The isolated nature of the Feldsher role provides the potential for further role development, and information technology improves the effectiveness of electronic health systems to support the use of diagnostic procedures.

*The academic arena*

Midwives can undergo further training to perform roles such as lecturer and researcher in maternal care. Roles such as lecturer-practitioner in the United Kingdom allow midwives to combine academic careers and retain clinical involvement.

The limitations identified in the preceding section also apply to the development in the academic arena. Furthermore, the knowledge and skills necessary for a midwife to attain the lecturer (or lecturer-practitioner) role require more than the planned 6-month duration of the postgraduate certificate courses^25^.

*The management arena*

Midwives can undertake further academic studies (e.g. MSc, MBA, or PhD) to develop the skills of leadership and control required to take roles such as Supervisor of Midwives or Head of Midwifery Services^27^. The limitations identified in the preceding section also apply to development in the management arena.

For a midwife to secure this position, they would have to undertake postgraduate studies in healthcare management. As entry is open to all midwives who have completed the Diploma in Midwifery Course or the BSc in Midwifery, participation in this course does not require additional training. It prepares a midwife to apply for the management position within a Clinical Unit. National leadership advocating for a clinical career path encompassing advanced practitioner roles is essential to address organizational culture. In addition to their clinical roles, midwives also work in education institutions, management, policy, research, regulation, midwives’ associations, and government. It is important to count and value midwives working in these areas, which are fundamentally important for the profession’s development^28^.

### Content requirements for the postgraduate certificate in midwifery

Postgraduate education for nurses and midwives in Mongolia is delivered with two forms of postgraduate training typically provided as: ‘specialized’ training carried out over 3 months and professional ‘advanced’ training, carried out over 1 month. The brevity of postgraduate training programs is inconsistent with postgraduate training programs typically delivered in developed economies. Nurses and midwives are required to have undertaken the ‘specialized’ training program before attending the 1-week ‘advanced’ program.

In many cases, undertaking postgraduate education appears to be driven by a requirement to meet the needs of credit accumulation rather than a constructive, planned building on undergraduate training. The fact that there are 15 providers offering such short ‘credit hours’ courses is a testament to this. The requirement to secure 3 credit points each year between each licensing period, suggests that the purpose of some courses is solely to meet the requirement of credit points and not personal professional development – the objective of continuous education^29^.

Above all, despite the importance of post-graduate education, the efficacy of these short courses has never been tested. Each educational program should be pre-validated to ensure that it delivers students sufficient educational benefits within the short time frame allotted for training. To overcome these difficulties, rigorous validation of post-graduate education is preemptively warranted to secure Mongolian nurses’ and midwives’ sustainable professional growth. A verification system is also necessary to monitor their completed credits and educational status.

Above all, curricula should keep up-to-date with international/national educational trends so that Mongolian nurses can be fully prepared to work with interdisciplinary/international collaborators and maximize their competencies in advanced health technologies. Finally, the postgraduate training period must be adjusted to last six months or more, combining theory and practical lessons, considered the minimum standard for quality education leading to professional improvement^30^. However, the recommendation to develop a ‘postgraduate certificate in midwifery’ has not been put forth, as there is little demand for such a program. A reasonable argument could be that the role of the Feldsher midwife is already advanced, although there is no ‘postgraduate’ training for this cohort. Also, given the isolated nature of their role and the widespread geographical locations of midwives in rural areas, it would be challenging to secure enough staff willing to attend a 6-month postgraduate certificate course in Ulaanbaatar.

Over the past few decades, midwifery has gained increased recognition, with the WHO advocating for the involvement of the most suitable practitioners in caring for women and babies during pregnancy, labor, birth, and the postnatal period in the absence of identified risk factors^31^. This article underscores the necessity of aligning midwifery education and practice with population health needs, including universal health coverage, and guides advancing the strengthening of midwifery.

It is important to note, however, that such educational policy initiatives may not directly address the shortage of midwives in actual clinical practice, given challenges like poor working conditions, low wages, excessive workloads, ineffective laws and regulations, and the burden of medical malpractice, among other factors^32^. Therefore, effectively addressing the midwifery shortage may necessitate advanced policy efforts that incorporate patient-centered precise nursing and midwifery, and utilize information technology, such as artificial intelligence, from various perspectives^33^.

Nevertheless, enhancing the quality of midwifery education in Mongolia and reshaping leadership has significant political implications for contributing substantially to the production of more highly qualified midwives in practice.

### Recommendations

This article described midwifery education and practice, including the role of midwives in the status of the professions in Mongolia. Although the World Health Organization has provided forty recommendations to enhance the professional status of midwifery, the lack of senior leadership in nursing and midwifery within the Ministry of Health has impeded the implementation of these recommendations. To enhance midwifery on a national scale, comprehensive leadership investments are crucial to support both educators and clinical practitioners, thereby guaranteeing the provision of high-quality care. The most important issue for strengthening midwifery is the need for continuing in-service training and postgraduate education. The potential for short courses to provide significant learning outcomes is questioned; however, it is acknowledged that several factors may influence the programs of training, including unsubstantiated quality of training, lack of midwifery workforce and well-prepared educators, and the widespread geographical locations of nurse-midwives across the country^34^. To address these challenges, we propose actionable and achievable recommendations as given Table 1.

**Table 1:** Policy recommendations and expected outcomes to improve the Mongolian midwifery professions

*Recommendations*	*Outcomes*
**Create a position of Chief Midwife**	The professions will have meaningful strategic leadership To assist with policy direction, technical advice, and support in the Ministry of Health Develop a Midwifery Scope of Practice, the National Midwifery Competencies, Professional Code of Practice for Midwives
**Develop new roles for midwives**	The role of the midwife (as defined by the ICM) does apply to midwives employed in tertiary services Midwifery will be recognized as an independent and science-based profession, no longer considered an assistant role to the obstetricians Management position will exit each level of service Establishing a clinical career path that encompasses the advanced practitioner’s roles Existing salary structure will be changed Organizational culture would be that the midwife-led model dominates Midwives’ engagement in decision-making, supervisory roles, and responsibilities for maternal and newborn care in practice will be enhanced Midwife’s roles will be well-defined
**Develop a strategic postgraduate training program for midwifery**	The professions will improve the educational level of midwives The midwife-led model dominates maternity care within tertiary hospitals The role of the midwife closely mirrors the international definition of the role of the midwife, with midwifery-led care more prevalent and the ‘scope of practice’ reflected in their activities Potential exists for further role development, e.g. utilizing information technology could provide effective e-health systems to support diagnostic procedures

## CONCLUSION

Based on our recommendations, the Mongolian government may pursue and strengthen the midwifery professions to secure quality maternal and newborn care and safety, easier health access, and better service delivery. Our recommendations will contribute significantly to developing effective and formal operational mechanisms for the Mongolian midwifery professions, and alleviating the midwifery shortage and inequality within the midwifery workforce.

## Data Availability

The data supporting this research are available from the authors on reasonable request.
